# An Underwater Image Denoising Method Based on High-Frequency Abrupt Signal Separation and Hybrid Attention Mechanism

**DOI:** 10.3390/s24144578

**Published:** 2024-07-15

**Authors:** Chunling Huo, Da Zhang, Huanyu Yang

**Affiliations:** 1 Changchun Institute of Optics, Fine Mechanics and Physics, Chinese Academy of Sciences, Changchun 130033, China; huochunling16@mails.ucas.ac.cn (C.H.); yanghuanyu16@mails.ucas.ac.cn (H.Y.); 2 University of Chinese Academy of Sciences, Beijing 100049, China; 3 Materials and Optoelectronics Research Center, University of Chinese Academy of Sciences, Beijing 100049, China

**Keywords:** underwater image denoising, convolutional neural network (CNN), attention mechanism, frequency domain decomposition

## Abstract

During underwater image processing, image quality is affected by the absorption and scattering of light in water, thus causing problems such as blurring and noise. As a result, poor image quality is unavoidable. To achieve overall satisfying research results, underwater image denoising is vital. This paper presents an underwater image denoising method, named HHDNet, designed to address noise issues arising from environmental interference and technical limitations during underwater robot photography. The method leverages a dual-branch network architecture to handle both high and low frequencies, incorporating a hybrid attention module specifically designed for the removal of high-frequency abrupt noise in underwater images. Input images are decomposed into high-frequency and low-frequency components using a Gaussian kernel. For the high-frequency part, a Global Context Extractor (GCE) module with a hybrid attention mechanism focuses on removing high-frequency abrupt signals by capturing local details and global dependencies simultaneously. For the low-frequency part, efficient residual convolutional units are used in consideration of less noise information. Experimental results demonstrate that HHDNet effectively achieves underwater image denoising tasks, surpassing other existing methods not only in denoising effectiveness but also in maintaining computational efficiency, and thus HHDNet provides more flexibility in underwater image noise removal.

## 1. Introduction

Underwater vision is a vital technology to explore the marine environment non-invasively, which could provide abundant and various information for ocean study. High-quality underwater images are essential for robots to complete underwater tasks such as exploration, archaeology, rescue, and imaging. However, underwater images are often distorted by water and suspended particles, which inevitably cause noise and reduce image usability. Well-denoised underwater images with high quality could assist scientific observations and robot underwater operations in working efficiently and accurately [1,2,3,4,5,6,7,8,9,10,11,12,13,14,15]. Furthermore, denoising technology could also support marine engineering by providing more precise and reliable data.

When light travels through water, its absorbance and scattering effect are influenced not only by water molecules but also by a combination of suspended particles such as sand grains, plankton, and dissolved organic matter. Consequently, the main challenges in underwater image denoising include low contrast, color distortion, and noise interference commonly observed in such images. To address these image quality issues, researchers have proposed several methods for underwater image restoration and enhancement over the past few decades. These methods [1,2,3,4,5] have significantly improved visibility and color correction in underwater images. Based on marine measurement data, Akkaynak et al. successfully derived the scattering space with physical effectiveness and constructed a revised underwater image generation formation model [1] to simulate the degradation process of underwater images. Similarly, in order to address the underwater image restoration problem, Desai et al. also designed a revised model and trained it with generative adversarial networks [2] to restore the real quality of underwater images. However, those two methods mentioned above still used RGB image inputs and did not consider separating and processing the noise component independently. From another aspect, Peng et al. tackled the challenge of separating color and texture in underwater images by proposing a U-shaped Transformer network [3] and introducing LAB and LCH color space to optimize the separation of color and texture, and they achieved significant results. Wang et al. observed inconsistencies in attenuation across different color channels and spatial regions in underwater images, thus leading to the development of a dual-information modulation network [4] to enhance the accuracy and robustness of underwater image restoration tasks. However, solely relying on color space for texture and color separation would be insufficient when dealing with underwater images. The reason is that image texture often contains both noisy and non-noisy components, and underwater image noise typically manifests as abrupt signal changes, which belong to the high-frequency part of the image. Failure to further separate these high-frequency signals during texture extraction can result in sub-optimal processing outcomes. Therefore, in addition to color space separation, further considerations are necessary from the perspective of frequency domain decomposition when processing underwater images. In this field, Li X et al. proposed the ACCE-D framework [5]. In the proposed framework, a Difference of Gaussian (DoG) filter and a bilateral filter were used to decompose the high-frequency and low-frequency components, respectively. Soft thresholding was then applied to suppress noise in the high-frequency components. Nevertheless, ACCE-D did not employ a learning-based denoising algorithm for training after separation and still left some progress to be made. The current underwater image denoising algorithms can be classified into two main categories: model-based methods [6,7,8,9,10,11,12,13,14,15] and learning-based methods [16,17,18,19,20,21,22,23,24,25,26,27,28,29,30]. Model-based methods remove noise from the image by modelling the noise distribution in the target image. Herein, filters designed manually are significant, such as bilateral filters [6], Gaussian filters, and median filters. The model-based method defines noise as abrupt signals with significant image gradients. By smoothing these abrupt signals, it can selectively remove noise from the target image. Additionally, wavelet transform thresholding-based denoising [7] is a commonly used technique in traditional image processing. It decomposes the signal into different scales and determines thresholds based on the energy of each scale in the way of setting low-energy wavelet coefficients to zero to achieve denoising. The non-local means (NLM) method [8] considers each pixel in the image and compares it with similar regions in other parts of the image. Different from traditional local denoising methods, NLM utilizes information from a wider area in the image, thereby better preserving details and structure. The block-matching and 3D filtering (BM3D) method [9] removes image noise by enhancing sparsity. Markov random field models [10] take each pixel in the image as a random variable, and model interactions between pixels by using an energy function and also find a configuration which could minimize the energy function to achieve denoising in the end. To simplify, model-based methods separate noise from images, suppress noise components, and then model noise removal. However, these methods carry the risk of losing image details. In addition, the performance of model-based methods may not be satisfying in complex scenarios in that they may struggle to remove various types of noise effectively.

Since the introduction of CNN algorithms like AlexNet [16] and ResNet [17], CNNs have been applied to image denoising tasks constantly [18,19,20,21,22,23,24,25,26]. DnCNN [18], proposed by Zhang K et al., was the first to apply CNNs to image denoising tasks, which defined a deep learning denoising equation as noisy images equal to clean images plus noise information to simulate the noise removal process. RIDNet [19], proposed by Anwar S et al., used residual structures to alleviate low-frequency information flow and feature attention to explore channel correlations. ECNDNet [20], proposed by Tian C et al., used dilated convolutions to enhance perception in the denoising process. ADNet [21], proposed by Liu Z et al., utilized sparse modules, feature enhancement modules, attention modules, and reconstruction modules to build a network structure for image denoising. MSANet [22], proposed by Gou Y et al., considered both intra-scale characteristics and cross-scale feature complementarity. SADNet [23], proposed by M Chang et al., introduced encoder and decoder blocks with context in capturing multi-scale information and removing noise ranging from coarse to fine. However, current CNNs cannot perceive long-distance interactions between pixels and also lack flexibility in learning and adjusting noise models, thus making CNNs less adaptable to different types and intensities of noise.

In recent years, some researchers have attempted to use Transformer architecture [27,28,29,30] for image denoising, as Transformers can capture long-distance interactions of pixels. Restormer [27] focuses on multi-scale local–global representation learning on high-resolution images. It introduces modules like Multi-Dconv Head Transposed Attention and Gated-Dconv Feed-Forward Network to aggregate locally and non-locally related pixels and control feature transformation. KBNet [28] combines the strengths of CNNs and Transformers and introduces the Kernel-Based Attention module to adaptively aggregate spatial neighborhood information, thereby using learnable kernels for different local patterns. Additionally, it also designs a separate lightweight convolution branch to predict linear combination coefficients for kernels, thus further enhancing the efficiency and performance of Transformer denoising. Therefore, combining lightweight convolutional networks with Transformers can improve the convergence speed of Transformers, making it easier to apply Transformers to low-level tasks such as image denoising.

In addition to improvements in network structures, researchers have also proceeded with denoising from the perspective of frequency domain separation [31,32,33,34,35,36]. From the frequency domain viewpoint, noise is primarily concentrated in the high-frequency signal region [31], which is characterized by sharp changes and is difficult to restore. Therefore, the approach involves using high–low frequency separation algorithms to divide the input image into high-frequency and low-frequency components. Denoising methods based on frequency domain separation include Fourier decomposition [32], wavelet decomposition [33], Laplacian high–low frequency decomposition [34], discrete cosine decomposition [35], and Gaussian blur decomposition [36]. CFPNet [35], proposed by Zhang K et al., employed discrete cosine decomposition to separate the image into high and low frequencies, and then processed these components individually using convolutional neural networks, thereby enhancing the ability to handle high-frequency signals. Wang L et al. used wavelet decomposition [33] to separate high and low frequencies and processed these components separately. However, methods like wavelet decomposition and discrete cosine decomposition are time-consuming and produce a large number of decomposed components. When using convolutional networks to learn from these extensive components, the computational load increases significantly. To reduce the time consumed by high–low frequency separation, Kang J et al. proposed the FSformer [36] image denoising network, which used a Gaussian blur kernel-based separation method. This method divided the input image into high- and low-frequency components, and reduced processing time effectively compared with wavelet decomposition. FSformer employed Transformer-based low-frequency (LFB) and high-frequency (HFB) modules to process the respective components separately, and then merged them to obtain the denoised image. While the aforementioned methods successfully separated high and low frequencies and addressed the issue of slow decomposition speeds, they did not differentiate the treatment of high- and low-frequency signals in their network structures, despite noise being primarily concentrated in the high-frequency signal region.

In recent research, some researchers have applied lightweight diffusion models to underwater image denoising tasks [37,38]. DM-Water [37], proposed by YI Tang et al., is a method that used diffusion models for image enhancement in underwater scenes. It generated corresponding enhanced images by using underwater images and Gaussian noise as input. Additionally, to improve the efficiency of the reverse process in diffusion models, they employed a lightweight Transformer-based denoising network to speed up both training and inference. WF-Diff [38], proposed by Chen Zhao et al., combined wavelet spatial frequency information of underwater images with diffusion models, which achieved state-of-the-art performance on several public datasets. However, diffusion models have the characteristic of generating tasks, making it difficult for the generated images to retain the original information of the underwater images. Moreover, diffusion models required significant computational resources.

To address the noise problem in underwater images, this paper proposes an algorithm called HHDNet, which is specialized to remove noise caused by environmental disturbances and technical constraints in the process of underwater robot photography, thereby improving the overall quality and clarity of images. Since noise in underwater images mainly concentrates on high-frequency abrupt signals, the HHDNet algorithm adopts a global residual learning approach. It decomposes RGB images into high-frequency and low-frequency components by using high–low frequency separation and utilizes a dual-branch network architecture to process high- and low-frequency parts independently. It also strengthens the perception and elimination of high-frequency abrupt noise during training. The contributions of this paper are as follows:

(1) We propose the HHDNet algorithm for underwater image denoising by targeting noise from environmental disturbances and technical limitations in underwater robot photography to enhance image quality and clarity. HHDNet adopts a Gaussian blur-based high–low frequency separation strategy and features a dual-branch network architecture.
(2) Compared to previous methods, HHDNet uses different modules in its dual-branch network based on the distinct characteristics of high and low frequencies. For high-frequency parts, it employs a Global Context Extractor (GCE) module, combining depthwise separable convolutions with a mixed attention mechanism to capture local details and global dependencies, focusing on removing abrupt noise. For low-frequency parts, it uses a computationally efficient residual convolution module to ensure precise and efficient noise removal.
(3) Compared to standard attention mechanisms and Transformers, the GCE module employs a mixed attention mechanism. To prevent convergence difficulties, a prior module with depthwise separable convolutions is introduced before the mixed attention mechanism. The inductive bias of convolutions assists the mixed attention mechanism to converge quickly during training, ensuring stronger denoising capabilities of HHDNet.

## 2. Underwater Image Denoising Network

**Figure 1:**
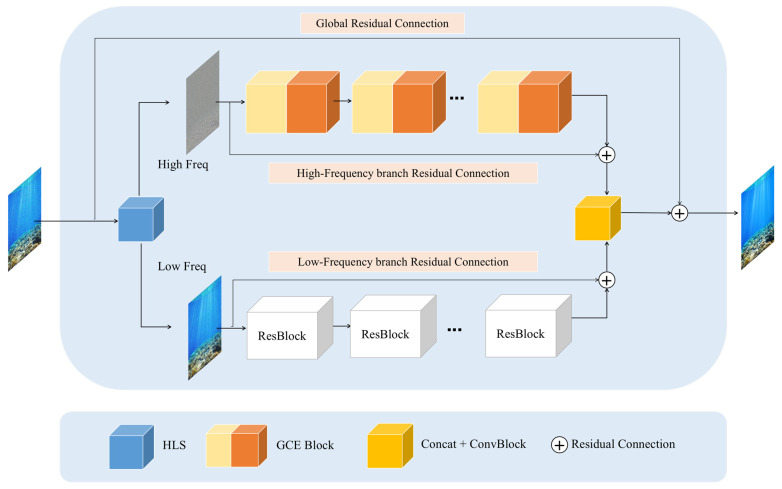
Structure of HHDNet.

Figure 1 shows the structure of the HHDNet, which adopts a dual-branch network architecture consisting of two branches. Each branch is constructed by stacking multiple cascaded feature extraction modules internally, enabling the deep extraction of various image features layer by layer and enhancing the network’s feature extraction capability. When processing images, the network firstly decomposes the degraded input image into high-frequency and low-frequency layers by using high–low frequency decomposition and then feeds them into the two branches for processing. The high-frequency branch uses eight GCE (Global Context Extractor) modules for high-frequency residual learning to remove high-frequency noise while preserving details. The low-frequency branch undergoes low-frequency residual learning through four residual convolution modules to restore the image’s basic structure. After learning, the residual features outputted by the high-frequency and low-frequency branches are added to the original layers, thus obtaining the denoised high-frequency and low-frequency information for precise reconstruction. Finally, the denoised layer information is concatenated, and a global residual amount is obtained by convolution fusion with a 3 × 3 filter, which is added to the original noisy image to obtain the clean image.

### 2.1. High–Low Frequency Separation

HHDNet uses Gaussian blur for high–low frequency decomposition to separate high-frequency and low-frequency information. Gaussian blur is an image processing technique used to reduce image noise and detail levels, resulting in a smoother image. By adjusting the values of Gaussian blur, the degree of blur for different frequency components in the image can be controlled. After applying Gaussian blur, the processed layer is combined or contrasted with the original layer in some form to extract high-frequency and low-frequency information, thus achieving high–low frequency separation. Assuming the input image is I, the Gaussian function is G, the mean of Gaussian noise is μ, and the variance is θ, the high–low frequency decomposition of the image can be represented as:

(1) $$LF=G_{\left(\theta ,\mu \right)}(I)$$

(2) HF=|I−LF|

As shown in Equations (1) and (2), the input RGB image undergoes Gaussian blur processing, and results in low-frequency information (LF). The high-frequency information (HF) is obtained by taking the absolute difference between the RGB image and the low-frequency information. High-frequency information typically corresponds to abrupt signals with significant gradients in the image, while low-frequency information represents the overall structure and colors of the image.

### 2.2. Global Context Extractor

In the high-frequency branch, eight cascaded Global Context Extractor (GCE) modules are utilized. The GCE module integrates a convolution group (ConvGroup) and cross-attention group, thereby enhancing the effectiveness of high-frequency image denoising. The role of the ConvGroup is to extract local features from the image and utilize bias induction to quickly identify and focus on areas with significant gradient changes in the image during the early stages of training. Furthermore, the cross-attention group has a more comprehensive long-distance perception and dependency capability, thus extracting global contextual information effectively. The GCE, constructed by combining the convolution group and cross-attention group, can selectively receive high-frequency images during training and process abrupt signals within them.

**Figure 2:**
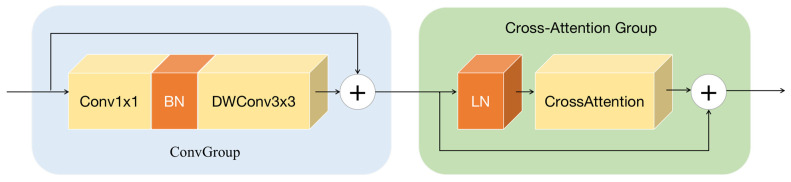
Structure of Global Context Extractor.

The GCE module is shown in Figure 2. During the construction, the feature map undergoes preliminary processing through a ConvGroup. The ConvGroup includes convolution layers, batch normalization (BN), and depthwise separable convolution (DWConv). The ConvGroup is defined as follows:

(3) ConvGroup(Z)=DWConv(BN(Conv(Z)))+Z

As shown in Equation (3), assuming the input feature is Z, it undergoes feature extraction using a 1 × 1 convolution operation first. Then, batch normalization (BN) is applied to normalize the feature map, enhancing the stability and convergence speed of the model. Next, a 3 × 3 depthwise separable convolution (DWConv) is used to further refine the features in order to reduce model complexity while maintaining high performance. The processed features are then added to the original input feature map to enable residual learning and alleviate the gradient vanishing problem during training of deep neural networks. 

After preliminary feature extraction in the convolution group, the output of the convolution group is passed into the cross-attention group. The cross-attention group consists of a layer normalization (LN) layer and a cross-attention module. LN is a normalization technique that normalizes the features across channels, providing stability during training. The cross-attention module facilitates information exchange between different parts of the input, allowing the model to focus on relevant areas for better performance in image denoising tasks.

**Figure 3:**
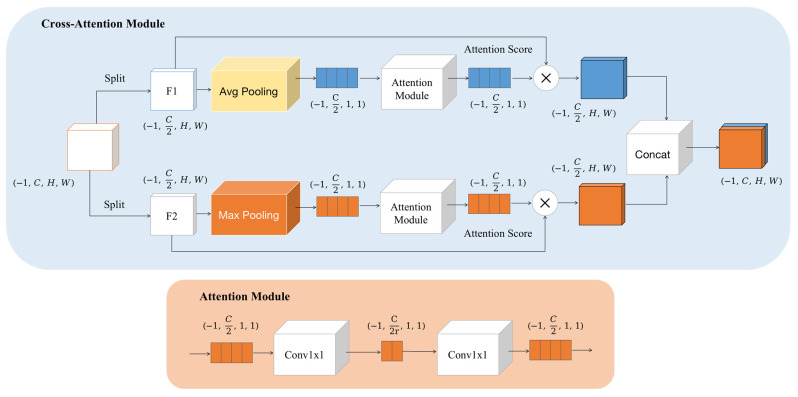
Structure of cross-attention module.

The cross-attention module is shown in Figure 3. After inputting feature map, the input is firstly split along the channel dimension to obtain two feature subsets, namely F1 and F2, both with half the number of channels of the original input. Different global pooling methods are applied to F1 and F2 for feature aggregation. F1 is processed through global average pooling to obtain mean information from all positions in the feature map, while F2 undergoes global max pooling. After pooling, F1 and F2 are compressed into feature vectors of size 1 × 1 × C/2. To further refine the feature representation, a strategy of dimensionality reduction followed by dimensionality expansion is utilized:

(4) $$d=Max(L,\frac{C}{2r})$$

As shown in Equation (4), d represents the number of channels after compressing either F1 or F2. The feature undergoes a 1 × 1 convolution operation and the number of channels in the feature vector is reduced to C/2r, where r is the dimension reduction factor. Subsequently, another 1 × 1 convolution layer is used to increase the number of channels back to C/2. After the dimensionality reduction and expansion operations, an attention score vector with the same number of channels as the input feature is obtained. Assuming the input is X and the attention score is A, the cross-attention module is defined as:

(5) $$CrossAtt(X)=Concat[F_{1}⨀{Att}_{1},F_{2}⨀{Att}_{2}]+X$$

As shown in Equation (5), the weighted feature representations are obtained by element-wise multiplication of Att1 and Att2 with the original F1 and F2, respectively. After concatenating the weighted F1 and F2 together, they are added to the input feature before channel splitting, serving as the output of the cross-attention mechanism module.

During training, F1 and F2 are cross-perceived and integrate information between different branches through cross-attention. The cross-attention module optimizes attention computation based on the module’s final output, ensuring that attention calculation maintains logicality and consistency while fully capturing and utilizing the complex features of the input data. It also explores the dependency relationships in the noisy regions from multiple perspectives.

### 2.3. Residual Block

The low-frequency component contains information such as color, saturation, and brightness, which are not included in the high-frequency component. This information collectively constitutes the basic color and overall perception of the image, and therefore plays an important role in underwater image denoising tasks. However, there is less noise information in the low-frequency part, so there is no need to use computationally intensive and structurally complex modules. This paper chooses to use low-complexity residual blocks [17] to construct the network structure for processing the low-frequency component, which can remove noise while preserving the original features of the low-frequency part.

**Figure 4:**
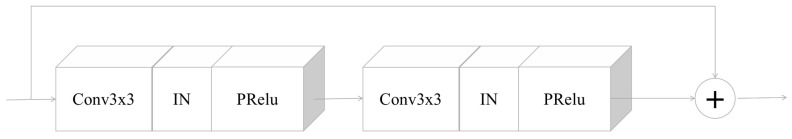
Structure of residual block.

The structure of the residual learning module is shown in Figure 4. It consists of two convolutional blocks which learn residual components through convolutional operations and then add themselves to the original components. Each convolutional block contains a 3 × 3 convolution, Instance Normalization [39] (IN), and Parametric Rectified Linear Unit (PRelu), respectively. IN is a normalization method that normalizes each channel of each input sample individually. PRelu is an activation function that improves upon the traditional ReLU function by introducing a learnable parameter to adaptively adjust the shape of the activation function in the negative region. The residual learning module can retain input information while learning and extracting more useful low-frequency feature representations.

### 2.4. Loss Function and Optimizer

Underwater image denoising based on deep learning uses a loss function to quantify the difference between actual values and predicted values. A smaller loss indicates better algorithm performance. In the training of HHDNet, image noise is defined as high-frequency abrupt signals. For the handling of high-frequency abrupt signals, this paper chooses the MAE loss function, also known as the L1 loss function, for supervision. As shown in Equation (6), where N represents the total number of training samples, $x_{i}$ represents the image after denoising by the network, and $y_{i}$ represents the true noise-free image:

(6) $$MAE=\frac{1}{N}\sum_{i=1}^{N}\left||x_{i}-y_{i}|\right|$$

Throughout the entire model training process, the optimizer plays a crucial role in facilitating parameter updates and guiding the model to its optimal state. The Adam optimizer combines the advantages of AdaGrad and RMSProp and leverages the strengths of both optimization algorithms. By comprehensively estimating the first and second moments of gradients, the Adam optimizer calculates the update step size. The simplicity of implementation and lower consumption in memory make Adam particularly suitable for models with large-scale data and parameters. Therefore, this paper chooses Adam to assist in achieving the best solution during model training.

## 3. Results and Discussion

### 3.1. Experimental Setup

The underwater data used in this experiment are drawn from the data source for the Underwater Robot Picking Competition (URPC) organized by the National Natural Science Foundation of China. The dataset used in this paper is URPC2019, consisting of images captured by underwater robots using cameras. The dataset contains 5543 images with a resolution of 640 × 480. The dataset is divided into training and testing sets in a 7:3 ratio. The training set includes 3880 ground truth images, while the testing set includes 1663 ground truth images. To train our HHDNet, Gaussian noise is added to the dataset at noise levels of 15, 25, and 50. The proposed HHDNet and comparison models are run on a single NVIDIA GeForce RTX 3090 graphics card. The HHDNet model is trained by using a partitioned original training dataset consisting of 64 × 64 input and output blocks. Training sessions are conducted separately for RGB color images with a fixed batch size of 16 and a learning rate set at 1 × 10^−3^. Data augmentation techniques are applied to enhance dataset diversity, including random vertical and horizontal flips, along with 90-degree rotations. Network parameter optimization during training is accomplished using the Adam optimizer.

### 3.2. Evaluation Metrics

In this paper, we used UCIQE, UIQM, PSNR, and SSIM to evaluate the performance of HHDNet. UCIQE and UIQM are primarily used for evaluating underwater image restoration tasks, while PSNR and SSIM are commonly used as metrics for image denoising tasks.

(1) The Underwater Color Image Quality Evaluation Index [40] (UCIQE) is a metric used for comprehensively evaluating the quality of color images. It evaluates color images from three aspects: the mean value of saturation, the standard deviation of hue, and the mean value of contrast. The larger the UCIQE value, the better the overall color quality of the image. The definition formula for this index is:
  (7) $$UCIQE=c_{1}\cdot \sigma_{c}+c_{2}\cdot \mu_{s}+c_{3}\cdot \sigma_{h}$$
  where $c_{1}$, $c_{2}$, and $c_{3}$ are weights assigned to these components based on their importance in the overall image quality evaluation, usually set as $c_{1}=$0.4680, $c_{2}=0$.2745, and $c_{3}=0$.2576. $\sigma_{c}$ is the standard deviation of contrast. $\mu_{s}$ is the mean value of saturation, and $\sigma_{h}$ is the standard deviation of hue.
(2) The Underwater Image Quality Measure index [41] (UIQM) is used to assess the quality of underwater images, focusing on three aspects: colorfulness, sharpness, and contrast. Colorfulness measures the naturalness and vividness of colors, contrast reflects the ability to distinguish objects and details in the image, and sharpness relates to the clarity of details and structures. By combining these factors, the UIQM index provides an evaluation of the overall quality of underwater images, where a higher value indicates better image quality. The formula for UIQM is typically given as:
  (8) $$UIQM=c_{1}\cdot UICM+c_{2}\cdot UISM+c_{3}\cdot UIConM$$

Underwater Image Colorfulness Measure (UICM) evaluates color richness and naturalness. Underwater Image Sharpness Measure (UISM) assesses image sharpness and clarity. Underwater Image Contrast Measure (UIConM) measures image contrast and distinction of objects. The UIQM index provides a quantitative measure of underwater image quality, crucial for assessing the effectiveness of image enhancement techniques in underwater images.

(3) The Peak Signal-to-Noise Ratio (PSNR) is used as an evaluation metric to measure the enhancement effect of HHDNet. Given the width and height of the input image as H and W, respectively, the enhanced image is denoted as $I_{c}$, and the original noisy image is denoted as In. The mean squared error (MSE) between the enhanced image and the original image is defined as:
  (9) $$MSE=\frac{1}{HW}\sum_{i=0}^{H-1}\sum_{j=0}^{W-1}{\left[I_{c}(i,j)-I_{n}(i,j)\right]}^{2}$$

The Peak Signal-to-Noise Ratio (PSNR) between the enhanced image and the original image is defined as:

(10) $$PSNR=10{log}_{10}(\frac{{MAX}_{I}}{MSE})$$

${MAX}_{I}$ represents the maximum pixel value of the image. If each pixel is represented by a B-bit binary number, then ${MAX}_{I}$ is equal to 2 raised to the power of B minus 1. In this paper, if each pixel is represented by an 8-bit binary number, then ${MAX}_{I}$ is 255.

(4) In addition, we also use the Structural Similarity Index [42] (SSIM) to measure the brightness, contrast, and structure (structural) between samples x and y.
  (11) $$l(x,y)=\frac{2\mu_{x}2\mu_{y}+c_{1}}{\mu_{x}^{2}{+\mu }_{y}^{2}+c_{1}}$$
  (12) $$c(x,y)=\frac{2\sigma_{x}2\sigma_{y}+c_{2}}{\sigma_{x}^{2}{+\sigma }_{y}^{2}+c_{2}}$$
  (13) $$s(x,y)=\frac{\sigma_{xy}+c_{3}}{\sigma_{x}\sigma_{y}+c_{3}}$$
  where $\mu_{x}$ and $\mu_{y}$ are the means of x and y, respectively; $\sigma_{x}$ and $\sigma_{y}$ are the variances of x and y, and $\sigma_{xy}$ is the covariance between x and y; and c1 and c2 are two constants. We set c3 = c2/2 to avoid being divided by zero. ${MAX}_{I}$ represents the maximum value of pixels in a B-bit image, which is 255 in this paper. By default, k1 = 0.01 and k2 = 0.03, and then we have:
  (14) $$SSIM(x,y)=[l{\left(x,y\right)}^{\alpha }{c(x,y)}^{\beta }{s(x,y)}^{\gamma }]$$

When α=β=γ=1, we have:

(15) $$SSIM(x,y)=\frac{\left(2\mu_{x}2\mu_{y}+c_{1})(2\sigma_{x}2\sigma_{y}+c_{2}\right)}{\left(\mu_{x}^{2}{+\mu }_{y}^{2}+c_{1})(\sigma_{x}^{2}{+\sigma }_{y}^{2}+c_{2}\right)}$$

### 3.3. Experimental Results

HHDNet employs a strategy of high–low frequency separation, utilizing a Gaussian blur-based approach for separation. Compared to other separation methods, Gaussian blur kernel high–low frequency separation is a real-time processing method. Table 1 provides an inference time comparison of Fourier decomposition, Wavelet decomposition, Laplacian decomposition, discrete cosine decomposition, and Gaussian blur decomposition.

HHDNet utilizes a high–low frequency decomposition strategy and also employs the GCE Block to process the high frequency. To validate the effectiveness of each improvement, this paper conducts ablation experiments. Firstly, the high–low frequency decomposition strategy is removed to verify its contribution to improving accuracy. Secondly, a comparison is made between the ResBlock and the GCE module in terms of accuracy improvement. Additionally, we incorporate inference time for each ablation experiment. Ultimately, when the low-frequency branch utilizes ResBlock and the high-frequency branch employs GCEBlock, the model achieves a good balance between accuracy and inference time. The results are shown in Table 2 and Table 3.

In HHDNet, high–low frequency decomposition is employed using Gaussian blur kernels. To determine the optimal Gaussian kernel size, we conduct the following experiments to compare the impact of different Gaussian kernels on UCIQE, UIQM, PSNR, and SSIM metrics, as shown in Table 4 and Table 5.

Observing at the same noise level, when Ksize increases from 3 × 3 to 5 × 5, UCIQE, UIQM, PSNR, and SSIM values all show improvement. However, as Ksize continues to increase to 7 × 7 and beyond, the improvement in metrics becomes very limited, and there is even a slight decrease in some cases. Therefore, this paper ultimately uses a Ksize of 5 × 5 as the parameter for the Gaussian kernel in the high–low frequency decomposition.

We conduct comparative experiments using ten methods, including NLM, BM3D, DnCNN-B, RIDNet, ECNDNet-L, ADNet-L, MSANet, SADNet, DM-Water, and WFI2-Diff. These ten methods are tested alongside our proposed HHDNet algorithm on the URPC2019 dataset. Ultimately, our algorithm outperforms other methods in terms of UCIQE, UIQM, PSNR, and SSIM in the URPC2019 testing, as shown in Table 6 and Table 7.

At a relatively low noise level with Sigma = 15, the proposed HHDNet algorithm achieves a UCIQE value of 0.631 and an UIQM value of 5.128. As the noise level increases to Sigma = 25, the UCIQE value of the HHDNet algorithm decreases to 0.598, with an UIQM value of 4.728. As the noise level increases to Sigma = 50, the UCIQE value of the HHDNet algorithm decreases to 0.557, with an UIQM value of 4.379.

At a relatively low noise level with Sigma = 15, the proposed HHDNet algorithm achieves a PSNR value of 31.554 and an SSIM value of 0.9421, showing significant advantages over other compared algorithms, indicating its effectiveness in restoring image quality and preserving structural information at this noise level. As the noise level increases to Sigma = 25, the PSNR value of the HHDNet algorithm decreases to 29.051, with an SSIM value of 0.9024, still surpassing other compared algorithms, demonstrating its stability and ability to preserve image structure across different noise levels. In the extreme case of high noise with Sigma = 50, although all algorithms experience a significant drop in SSIM values, HHDNet still achieves a PSNR value of 26.005 and an SSIM value of 0.8248 and shows its capability to recover images and preserve structure even under extremely high noise levels.

The total number of model parameters (Parameters), model computational complexity (FLOPs), and inference time to some extent reflect the model’s complexity. If the total amount of model parameters and computational complexity is too high, the model may not be suitable for practical applications. Therefore, to validate the rationality of the model, as shown in Table 8, the total amounts of model parameters, computational complexity, and Inference Time for each algorithm are calculated. From the table, it can be seen that our model’s total amounts of parameters and computational complexity are relatively reasonable. This model can effectively remove image noise in practical applications.

HHDNet demonstrates significant advantages in performance. Compared with other methods, HHDNet achieves a superior balance between speed and accuracy. HHDNet has 17.5 G flops and 6.82 M parameters, and an inference time of 16.3 ms, which strikes a balance between computational efficiency and model complexity by avoiding being excessively large, causing low computational efficiency, or too small, limiting model complexity.

**Figure 5:**
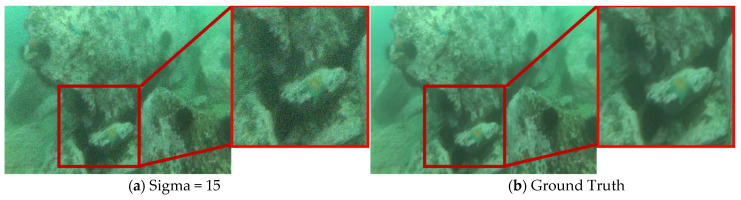

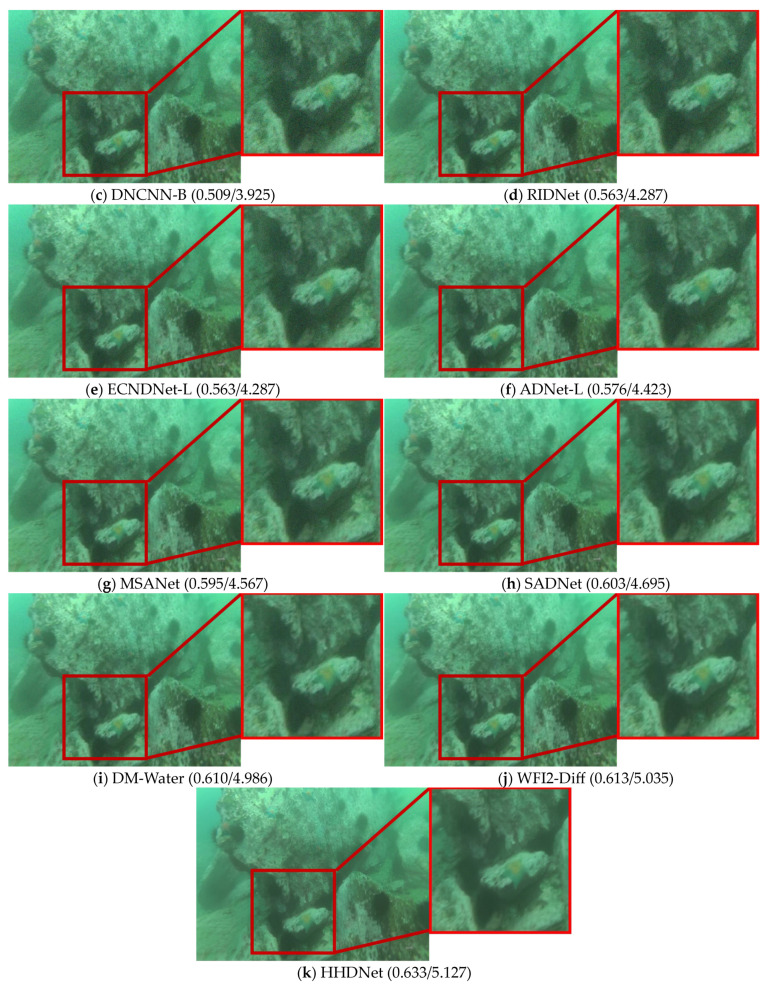
When Sigma = 15, select an image from the URPC2019 test set and denoise it by using different algorithms.

**Figure 6:**
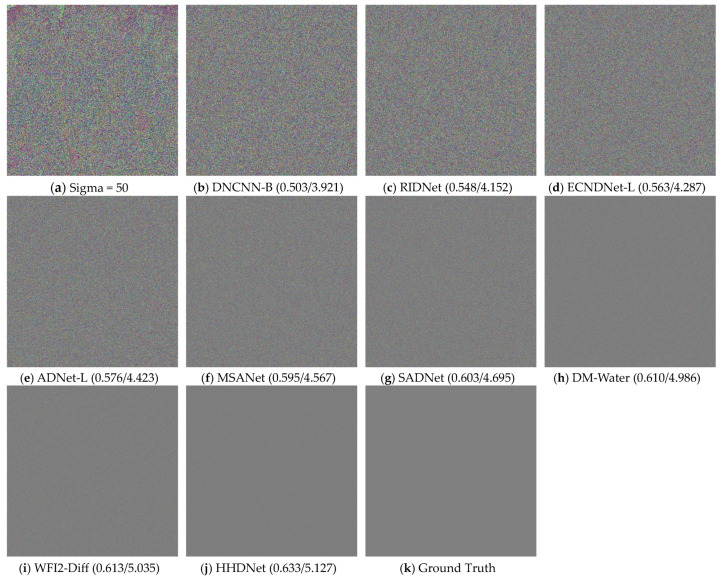
When Sigma = 15, visualize the error map (difference and the distribution of error between the prediction and the ground truth). Then, select the image of Figure 6 and denoise it using different algorithms.

**Figure 7:**
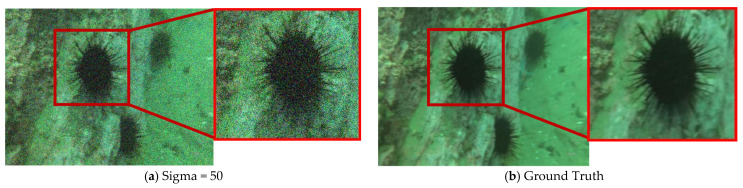

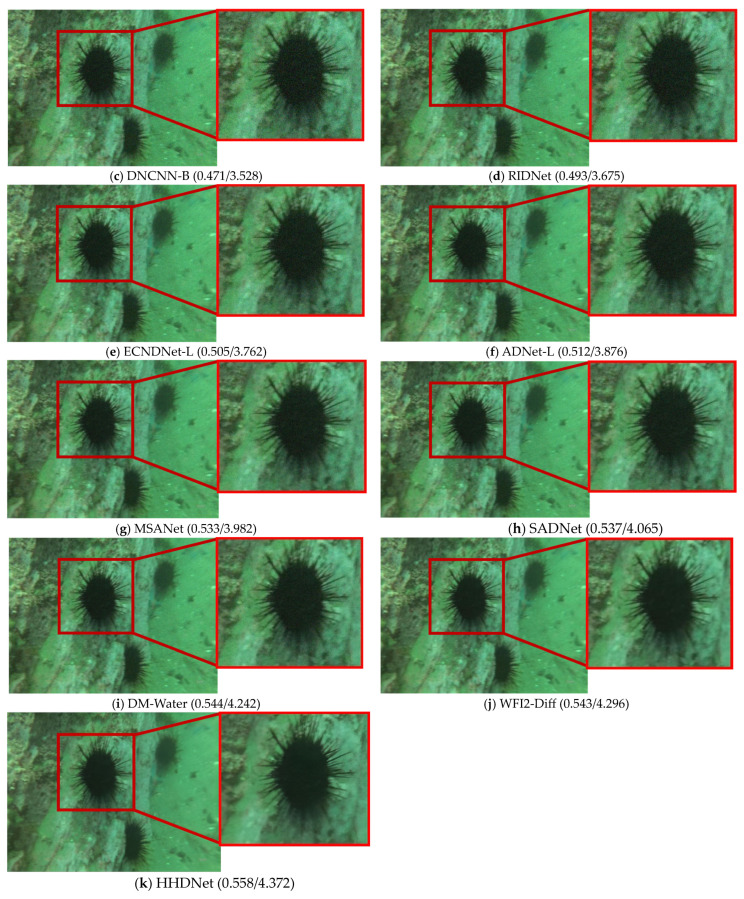
When Sigma = 50, select an image from the URPC2019 test set and denoise it by using different algorithms.

**Figure 8:**
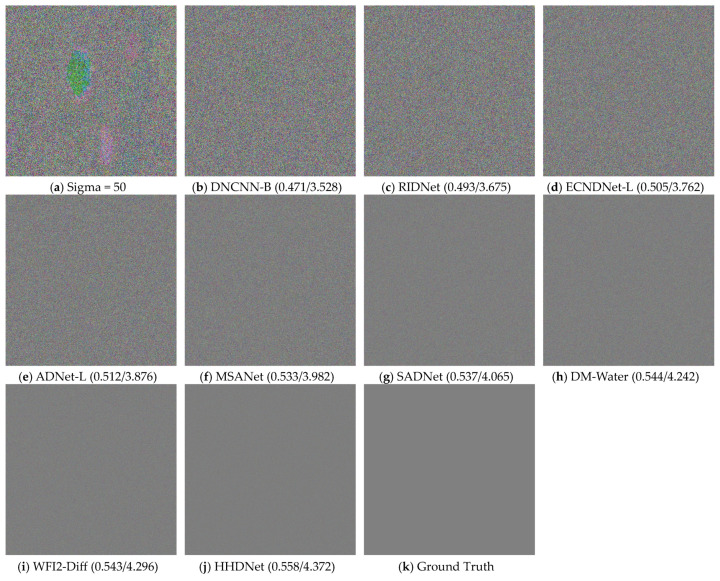
When Sigma = 50, visualize the error map (difference and the distribution of error between the prediction and the ground truth). Then, select the image of Figure 7 and denoise it using different algorithms.

To demonstrate the superiority of our proposed HHDNet, we select an image from the URPC2019 dataset and compare our denoising results with those of other algorithms. We visualize images with noise levels of 15 and use error maps to display them in Figure 5 and Figure 6. Similarly, we visualize images with noise levels of 50 and use error maps to display them in Figure 7 and Figure 8.

(16) $$ErrorMap=\frac{\sigma }{N}\sum_{i=1}^{N}\left||x_{i}-y_{i}|\right|+\mu$$

Among them, setting σ = 5, μ = 128 when Sigma = 15, and setting σ = 3, μ = 128 when Sigma = 50, can make the error map more intuitive.

These images clearly indicate that the denoising results produced by our algorithm are significantly clearer and preserve image details effectively. Additionally, both UCIQE and UIQM metrics are higher.

From the visualization results, the HHDNet algorithm demonstrates good structural preservation performance under both low and high noise levels, especially in low to moderate noise levels, where it performs exceptionally well. Compared with other algorithms, HHDNet still achieves relatively high UCIQE and UIQM values.

## 4. Conclusions

This paper proposes an underwater image denoising algorithm named HHDNet. The algorithm adopts a dual-branch network architecture for high- and low-frequency components and integrates a hybrid-attention GCE module to enhance and accurately identify high-frequency noise spike signals, thus effectively removing noise generated during underwater robot photography due to complex environments and technical limitations. It not only surpasses existing methods in denoising performance on the URPC2019 dataset but also demonstrates significant advantages in computational efficiency, performing underwater image denoising more precisely and efficiently. The proposed method improves the visual quality of underwater image denoising significantly and could contribute to visual-based underwater tasks such as subsequent underwater detection and segmentation tasks.

As underwater scientific research and industrial applications develop further, the demand for high-quality underwater images is becoming increasingly urgent. HHDNet could bring significant improvement in underwater image denoising technology and provide strong support for further development. In particular, HHDNet demonstrates notable advantages in computational efficiency, thereby enhancing resource usage efficiency. The outstanding denoising effect and lower resource consumption make HHDNet absolutely predominant among competitors in completing tasks such as target detection and image segmentation. We strongly believe that HHDNet would bring new breakthroughs and practical value to the development of underwater image processing.

Nonetheless, HHDNet still has room for improvement. The formation of underwater image noise is complex and diverse, with significant variances in the distribution of different noise types. In practical applications, denoising models need to map the noise domain containing multiple types of noise to the high-quality image domain, which is essentially a many-to-many task. It is confined to the current supervised training using only Euclidean distance, which may lead to the training process converging to an average level. To further enhance denoising effectiveness, we could consider exploring advanced techniques such as adversarial networks or diffusion models to address this issue and promote the continuous advancement of underwater image processing technology.

## Figures and Tables

**Table 1:** Inference time comparison of Fourier decomposition, Wavelet decomposition, Laplacian decomposition, discrete cosine decomposition, and Gaussian blur decomposition. Bold represents the shortest time.

	Fourier Decomposition	Wavelet Decomposition	Laplacian Decomposition	Discrete Cosine Decomposition	Gaussian Blur Decomposition
**Inference Time**	26.87 ms	7.25 ms	3.40 ms	11.85 ms	**0.92 ms**

**Table 2:** HHDNet’s ablation experiments in terms of average UCIQE and UIQM at noise levels 15, 25, and 50. **×** represents High-Low Frequency Decomposition is not used, √ represents High-Low Frequency Decomposition is used, and bold represents the configuration used by HHDNet.

High–Low Frequency Decomposition	Low-Frequency Branch	High-Frequency Branch	Inference Time	Sigma = 15		Sigma = 25		Sigma = 50	
				UCIQE	UIQM	UCIQE	UIQM	UCIQE	UIQM
**×**	**ResBlock (RGB Input)**		6.2 ms	0.529	4.212	0.517	4.115	0.488	3.756
√	ResBlock	ResBlock	9.3 ms	0.573	4.727	0.551	4.456	0.529	4.118
√	ResBlock	GCEBlock	**16.3 ms**	**0.631**	**5.128**	**0.598**	**4.728**	**0.557**	**4.379**
√	GCEBlock	GCEBlock	26.2 ms	0.638	5.142	0.605	4.737	0.566	4.388

**Table 3:** HHDNet’s ablation experiments in terms of average PSNR and SSIM at noise levels 15, 25, and 50. **×** represents High-Low Frequency Decomposition is not used, √ represents High-Low Frequency Decomposition is used, and bold represents the configuration used by HHDNet.

High–Low Frequency Decomposition	Low-Frequency Branch	High-Frequency Branch	Sigma = 15		Sigma = 25		Sigma = 50	
			PSNR	SSIM	PSNR	SSIM	PSNR	SSIM
**×**	**ResBlock (RGB Input)**		31.541	0.9406	29.015	0.9015	25.988	0.8238
√	ResBlock	ResBlock	31.552	0.9418	29.043	0.9022	26.002	0.8246
√	ResBlock	GCEBlock	**31.554**	**0.9421**	**29.051**	**0.9024**	**26.005**	**0.8248**
√	GCEBlock	GCEBlock	31.554	0.9422	29.052	0.9024	26.005	0.8249

**Table 4:** HHDNet’s high–low frequency decomposition with different Gaussian kernel parameters in terms of average UCIQE and UIQM at noise levels 15, 25, and 50. Bold represents the configuration used by HHDNet.

Gaussian Ksize	Sigma = 15		Sigma = 25		Sigma = 50	
	UCIQE	UIQM	UCIQE	UIQM	UCIQE	UIQM
3 × 3	0.622	5.023	0.583	4.634	0.549	4.323
**5 × 5**	**0.631**	**5.128**	**0.598**	**4.728**	**0.557**	**4.379**
7 × 7	0.619	5.077	0.586	4.663	0.544	4.298
9 × 9	0.601	4.915	0.572	4.578	0.537	4.216
11 × 11	0.596	4.823	0.565	4.423	0.529	4.169

**Table 5:** HHDNet’s high–low frequency decomposition with different Gaussian kernel parameters in terms of average PSNR and SSIM at noise levels 15, 25, and 50. Bold represents the configuration used by HHDNet.

Gaussian Ksize	Sigma = 15		Sigma = 25		Sigma = 50	
	PSNR	SSIM	PSNR	SSIM	PSNR	SSIM
3 × 3	31.553	0.9412	29.047	0.9017	25.999	0.8244
5 × 5	**31.554**	**0.9421**	**29.051**	**0.9024**	**26.005**	**0.8248**
7 × 7	31.549	0.9420	29.048	0.9022	26.004	0.8248
9 × 9	31.548	0.9411	29.045	0.9019	26.002	0.8241
11 × 11	31.545	0.9408	29.044	0.9015	26.000	0.8239

**Table 6:** The average UCIQE and UIQM values of different algorithms at noise levels 15, 25, and 50 on the URPC2019 test set. Bolded “HHDNet” represents the algorithm proposed in this paper, and bolded metrics represents the best results in the comparative experiments.

Method	Sigma = 15		Sigma = 25		Sigma = 50	
	UCIQE	UIQM	UCIQE	UIQM	UCIQE	UIQM
NLM [8]	0.318	2.713	0.284	2.472	0.259	2.194
BM3D [9]	0.336	2.829	0.297	2.541	0.266	2.302
DnCNN-B [18]	0.503	3.921	0.509	3.841	0.472	3.527
RIDNet [19]	0.543	4.150	0.522	3.935	0.491	3.672
ECNDNet-L [20]	0.561	4.289	0.537	4.016	0.505	3.764
ADNet-L [21]	0.578	4.421	0.550	4.123	0.518	3.875
MSANet [22]	0.592	4.568	0.564	4.239	0.533	3.981
SADNet [23]	0.604	4.697	0.575	4.312	0.538	4.068
DM-Water [37]	0.609	4.987	0.582	4.563	0.543	4.241
WFI2-Diff [38]	0.612	5.032	0.585	4.617	0.545	4.298
**HHDNet**	**0.631**	**5.128**	**0.598**	**4.728**	**0.557**	**4.379**

**Table 7:** The average PSNR and SSIM values of different algorithms at noise levels 15, 25, and 50 on the URPC2019 test set. Bolded “HHDNet” represents the algorithm proposed in this paper, and bolded metrics represents the best results in the comparative experiments.

Method	Sigma = 15		Sigma = 25		Sigma = 50	
	PSNR	SSIM	PSNR	SSIM	PSNR	SSIM
NLM [8]	29.412	0.8734	26.699	0.8472	22.8873	0.7851
BM3D [9]	29.641	0.8828	27.128	0.8594	22.8964	0.7899
DnCNN-B [18]	31.540	0.9406	29.016	0.9016	25.988	0.8238
RIDNet [19]	31.542	0.9420	29.042	0.9021	25.994	0.8242
ECNDNet-L [20]	31.546	0.9414	29.038	0.9014	25.991	0.8240
ADNet-L [21]	31.548	0.9410	29.033	0.9017	25.997	0.8243
MSANet [22]	31.551	0.9414	29.045	0.9022	26.001	0.8246
SADNet [23]	31.553	0.9412	29.047	0.9023	26.000	0.8247
DM-Water [37]	31.532	0.9414	29.040	0.9017	25.990	0.8241
WFI2-Diff [38]	31.549	0.9418	29.048	0.9022	26.001	0.8245
**HHDNet**	**31.554**	**0.9421**	**29.051**	**0.9024**	**26.005**	**0.8248**

**Table 8:** The comparison of model parameters and flops between the HHDNet method and other algorithms, with all algorithms evaluated on a single RTX 3090 GPU.

	DnCNN-B	RIDNet	ECNDNet-L	ADNet-L	MSANet	SADNet	DM-Water	WFI2-Diff	HHDNet
Flops	23.5 G	21.4 G	34.5 G	35.7 G	27.1 G	45.2 G	150.6 G	631.1 G	17.5 G
Parameters	12.78 M	6.79 M	15.2 M	15.8 M	7.99 M	17.29 M	10.13 M	43.72 M	6.82 M
Inference Time	34 ms	19.8 ms	79.1 ms	79.8 ms	25.3 ms	42.2 ms	130 ms	720.4 ms	16.3 ms

## Data Availability

The underwater image data that support the findings of this study are openly available at https://universe.roboflow.com/underwater-fish-f6cri/urpc2019-nrbk1 (accessed on 1 December 2023).
